# Inverse Tunnel Magnetocapacitance in Fe/Al-oxide/Fe_3_O_4_

**DOI:** 10.1038/s41598-017-02361-4

**Published:** 2017-06-01

**Authors:** Hideo Kaiju, Taro Nagahama, Shun Sasaki, Toshihiro Shimada, Osamu Kitakami, Takahiro Misawa, Masaya Fujioka, Junji Nishii, Gang Xiao

**Affiliations:** 1 Research Institute for Electronic Science, Hokkaido University, Sapporo, Hokkaido 001-0020 Japan; 2 Graduate School of Engineering, Hokkaido University, Sapporo, Hokkaido 060-8628 Japan; 3 Institute of Multidisciplinary Research for Advanced Materials, Tohoku University, Sendai, Miyagi 980-8577 Japan; 4 Department of Physics, Brown University, Providence, RI 02912 USA

## Abstract

Magnetocapacitance (MC) effect, observed in a wide range of materials and devices, such as multiferroic materials and spintronic devices, has received considerable attention due to its interesting physical properties and practical applications. A normal MC effect exhibits a higher capacitance when spins in the electrodes are parallel to each other and a lower capacitance when spins are antiparallel. Here we report an *inverse tunnel magnetocapacitance* (TMC) effect for the first time in Fe/AlO_x_/Fe_3_O_4_ magnetic tunnel junctions (MTJs). The inverse TMC reaches up to 11.4% at room temperature and the robustness of spin polarization is revealed in the bias dependence of the inverse TMC. Excellent agreement between theory and experiment is achieved for the entire applied frequency range and the wide bipolar bias regions using Debye-Fröhlich model (combined with the Zhang formula and parabolic barrier approximation) and spin-dependent drift-diffusion model. Furthermore, our theoretical calculations predict that the inverse TMC effect could potentially reach 150% in MTJs with a positive and negative spin polarization of 65% and −42%, respectively. These theoretical and experimental findings provide a new insight into both static and dynamic spin-dependent transports. They will open up broader opportunities for device applications, such as magnetic logic circuits and multi-valued memory devices.

## Introduction

Magnetocapacitance (MC) effect has attracted much attention due to their fascinating spin phenomena, such as spin capacitance^1–4^, frequency-dependent spin transport^5–8^ and potential applications^7, 9–11^ as highly-sensitive magnetic sensors, high-frequency devices and energy storage materials. The MC effect has been observed in multiferroic materials^12–15^, spintronic devices^3, 6–10, 16–20^ and magnetic supercapacitors^11, 21^. Here, the spintronic devices include magnetic tunnel junctions (MTJs)^8^, molecular spin valves (SVs)^19^, magnetic nanogranular (MNG) films^7^ and magnetic single-electron transistors (SETs)^20^. The MC effect observed in magnetic tunneling systems is generally referred to as tunnel magnetocapacitance (TMC), for example, in AlO_*x*_- and MgO-based MTJs^6, 8, 10^, Fe_9_Co_8_/Mg_26_F_57_ MNG films^7^ and Co/Al_2_O_3_/Al/Ni_80_Fe_20_/Al/Al_2_O_3_/Co SETs^20^.

In TMC devices, the capacitance depends on the relative spin directions of the two electrodes. When the spins in both magnetic electrodes are parallel to each other, to be referred to as the parallel (P) configuration, the value of *C*_P_ is larger than the value of *C*_AP_ in the antiparallel (AP) configuration (which also corresponds to the random spin state for MNG films). This phenomenon can be explained by the Debye-Fröhlich (DF) model for the dynamic spin behavior^7, 8, 22^, and by the spin-dependent drift-diffusion (SDD) model for the static case^20^. Namely, the DF model provides adequate description to the frequency response of the dipoles, formed by the electrons and holes within the ferromagnet/insulator (FM/I) interfacial region. Due to the fact that the relaxation time *τ*_P_ in P state is larger than the *τ*_AP_ in AP state, the DF model reveals a larger dynamic dielectric polarization in P state than that in AP state. In the static case, the diffusion length differs between the majority and the minority spins, as a result of the accumulation of minority spins and the depletion of majority spins within the interfacial regions in the AP state. This difference forms a tiny charge dipole, which gives rise to an additional serial capacitance, resulting in reduced *C*_AP_. We herein note that the observation of such “normal” TMC (i.e., *C*_P_ > *C*_AP_) is essentially attributed to the electronic states near the interfacial Fermi levels, which determine the relaxation time and the diffusion length. This suggests that a new phenomenon of an *inverse TMC* (i.e., *C*_P_ < *C*_AP_) could be observed by modulating the density of states (DOS) of the ferromagnetic materials. The observation of the inverse TMC will open up new opportunities for device applications, such as magnetic logic circuits, impedance-tunable spin filters and multi-valued memory devices.

One way to observe the inverse TMC is to use FM_1_/I/FM_2_ MTJs, in which the sign of spin polarization *P*_1_ of the ferromagnetic layer FM_1_ is opposite to that of the *P*_2_ of FM_2_; for example, when Fe (*P*_1_ > 0) is used as FM_1_, another material with *P*_2_ < 0 should be used as FM_2_. In this situation, the tunneling probability for P state is smaller than that in AP state, enabling the inversion of the relaxation time as well as the diffusion length. Therefore, the materials showing negative spin polarization is required to find the inverse TMC. Earlier extensive studies have led to the discovery of a few FM solids with negative spin polarization, such as Fe_3_O_4_^23, 24^, Fe_4_N^25^ and SrRuO_3_^26^. In particular, Fe_3_O_4_ is one of the most promising solids with negative spin polarizations^23, 24, 27–31^, because of its high Curie temperature of 858 K and a good electrical conductivity of ~250 ${{\rm{\Omega }}}^{-1}{{\rm{cm}}}^{-1}$. The *P* of Fe_3_O_4_ has been measured as −16% at room temperature^24^ and −32% at 70 K^23^. The first-principle band calculation also predicts that the Fe_3_O_4_ could be a half metal with a *P* of −100%^27–29^.

In this work, we report the first observation of an *inverse TMC effect* using Fe/AlO_*x*_/Fe_3_O_4_ MTJs at room temperature. The inverse TMC reaches up to 11.4% and the robustness of spin polarization is revealed in the bias dependence of the inverse TMC. The frequency characteristics and bias dependence of the inverse TMC can be well explained by a newly proposed theoretical calculation based on DF model (combined with Zhang formula and parabolic barrier approximation) and SDD model. Based on this calculation, we predict that the inverse TMC could reach 150% in MTJs with a positive and negative spin polarization of 65% and −42% for the two electrodes, respectively.

## Results and Discussion

### Device structure

**Figure 1:**
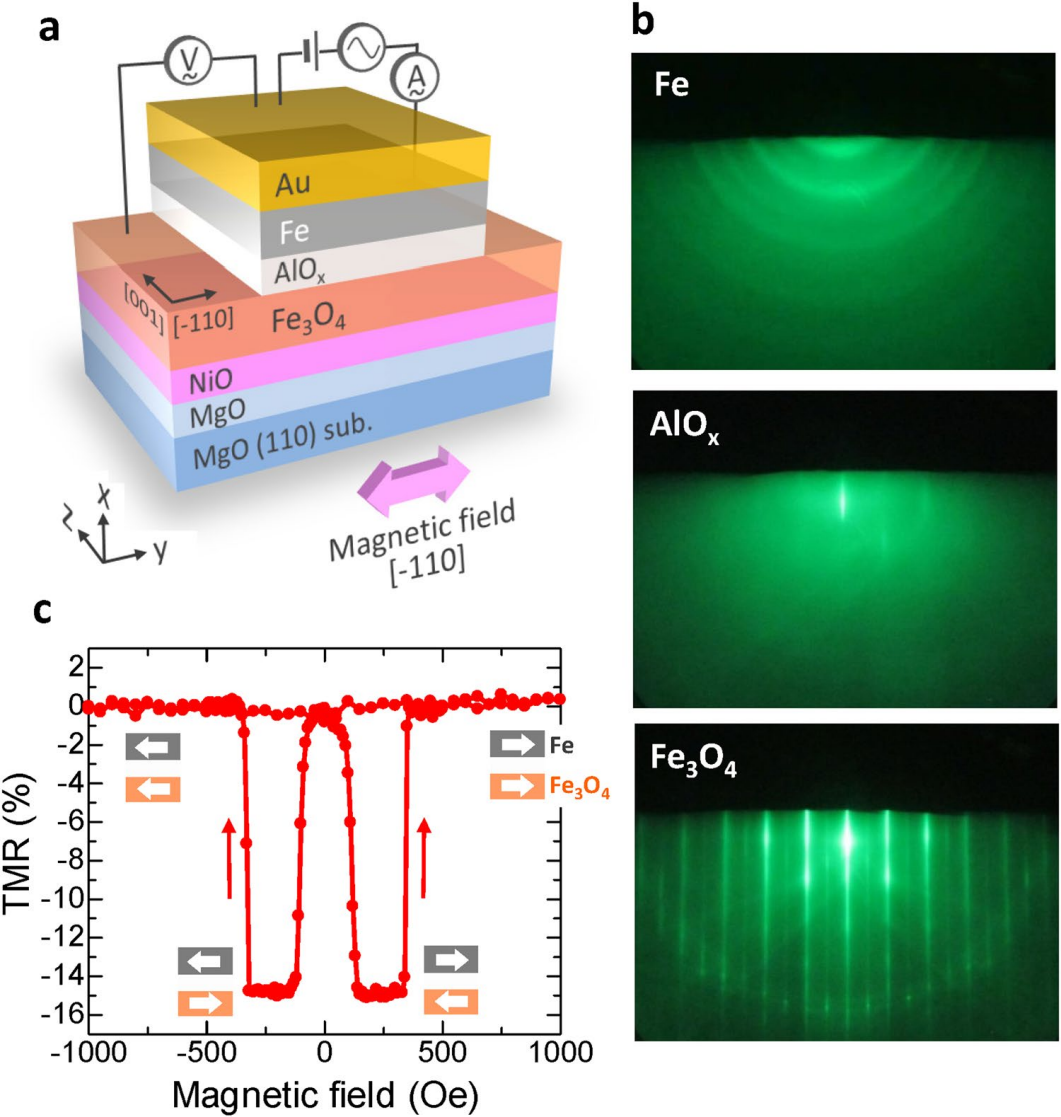
Experimental set-up and device structure. (**a**) Schematic of an Fe_3_O_4_-based MTJ, with the structure: MgO(110)/MgO(20 nm)/NiO(5 nm)/Fe_3_O_4_(60 nm)/AlO_*x*_(2–4 nm)/Fe(10 nm)/Au(30 nm). For patterning the MTJ, a photolithography, Ar ion-milling process and SiO_2_ sputtering were used. The measurement set-up for inverse TMC is also shown. The magnetic field is applied along the [−110] direction. (**b**) RHEED patterns of Fe, AlO_x_ and Fe_3_O_4_. (**c**) TMR curve measured by a dc four probe method, showing a negative TMR of −15%.

Figure 1a shows the device structure prepared by a molecular beam epitaxy (MBE) system, in a chamber with a base pressure of 10^−8^ Pa, with the following layer sequence: MgO(110)/MgO(20 nm)/NiO(5 nm)/Fe_3_O_4_(60 nm)/AlO_*x*_(2–4 nm)/Fe(10 nm)/Au(30 nm). Details of the device fabrication procedure are described in the Experimental Section. The crystallinity of the films was evaluated by reflection high energy electron diffraction (RHEED). Figure 1b shows the RHEED patterns of Fe, AlO_*x*_ and Fe_3_O_4_ layers. The electron beam was incident along the [001] direction. A clear streak pattern can be observed for Fe_3_O_4_, indicating that the films are epitaxially grown. The RHEED patterns also reveal that AlO_*x*_ is amorphous and Fe is a polycrystalline structure. After the MBE growth of the multilayer stack, the MTJ structures with a junction area of 10 × 10 μm^2^ were patterned by using standard photolithography with Ar ion-milling and SiO_2_ insulation overlayer.

The current-voltage (*I-V*) characteristics and tunnel magnetoresistance (TMR) curves in MTJs were measured by a dc four-probe method at room temperature. A typical TMR curve is shown in Fig. 1c. A clear inverse TMR effect can be observed; the resistance *R*_P_ in the P configuration is larger than the *R*_AP_ in the AP configuration. The TMR exhibits −15%, which is the highest value ever reported for MTJs with an AlO_*x*_ barrier and Fe_3_O_4_ electrodes at room temperature. In this study, three types of MTJs, showing TMR of −1% (sample A), −7% (sample B) and −15% (sample C), respectively, were prepared.

### Modeling of the inverse TMC

The calculation of the frequency characteristics and bias dependence of the inverse TMC is performed using modified DF and SDD models. The DF model is a useful tool for the calculation of dynamic complex dielectric constant in a variety of insulating solid and liquid systems under the equilibrium state^22, 32–35^. This model can be applied to the inverse TMC in MTJs, i.e., the capacitance ${C}_{{\rm{P}}({\rm{AP}})}^{{\rm{DF}}}(f)$ as a function of frequency *f* for the P(AP) configuration in MTJs can be expressed by Supplementary equation (2). In the presence of the bias voltage, the non-equilibrium term should be taken into account in DF model. In our calculation, not only Julliere formula^36^ but also Zhang model^37^ is incorporated for the calculation of the relaxation time appeared in DF model. The relaxation time in this model is related to the motion of electric dipoles, formed by electrons and holes near the FM/I interfaces. The detailed explanation on the relaxation time has been described in our previous paper^8^. Moreover, since the effective barrier thickness, contributing to the measured capacitance, can be changed by the bias voltage, the parabolic barrier approximation is used to determine the barrier thickness. The detailed derivation is described in the Supplementary Information section. As a result of the formulation, the capacitance ${C}_{{\rm{P}}({\rm{AP}}),V}^{\text{DF}-\text{ZP}}(f)$ with applied bias voltage *V* for the P (AP) configuration, based on the DF model combined with Zhang formula and the parabolic barrier approximation, can be expressed by

1 $$\begin{array}{rcl}{C}_{{\rm{P}}({\rm{AP}}),\,V}^{{\rm{DF}}-{\rm{ZP}}}(f) & = & \frac{1}{1-e(1-\gamma )V/4{\phi }_{0}}\,[{C}_{\infty ,{\rm{P}}({\rm{AP}})}\,+\,\frac{{C}_{0,{\rm{P}}({\rm{AP}})}-{C}_{\infty ,{\rm{P}}({\rm{AP}})}}{2}\\  &  & \times (1-\frac{\sinh \,[{\beta }_{{\rm{P}}({\rm{AP}})}\,\mathrm{ln}(2\pi f{\tau }_{{\rm{P}}({\rm{AP}}),V})]}{\cosh \,[{\beta }_{{\rm{P}}({\rm{AP}})}\,\mathrm{ln}(2\pi f{\tau }_{{\rm{P}}({\rm{AP}}),V})]+\,\cos ({\beta }_{{\rm{P}}({\rm{AP}})}\pi /2)})],\end{array}$$

where *e* is the electron charge, *γ* is a parameter determining the effective applied voltage (0 < *γ* < 1) and *ϕ*_0_ is the barrier height in the absence of the bias voltage. *C*_∞, P(AP)_ and *C*_0, P(AP)_ are the high-frequency and static capacitances, *τ*_P(AP)_, _*V*_ is the relaxation time at *V* and *β*_P(AP)_ is the exponent showing the distribution of relaxation time (0 < *β* < 1), respectively, for the P(AP*)* configuration. The relaxation time can be given by

2 $${\tau }_{{\rm{P}}({\rm{AP}}),V}=\frac{1}{1+{K}_{{\rm{P}}({\rm{AP}})}(1-\gamma )V}{\tau }_{{\rm{P}}({\rm{AP}}),0},$$

where ${K}_{{\rm{P}}({\rm{AP}})}$ is a parameter determined by Curie temperatures of FM_1_ and FM_2_, the DOS of itinerant electrons in FM_1_ and FM_2_, and direct and spin-dependent transfers and spin quantum number within the framework of the transfer Hamiltonian in the system of FM_1_/I/FM_2_. The ${K}_{{\rm{P}}({\rm{AP}})}$ is assumed to be an adjustable parameter in our calculation.

The capacitance ${C}_{{\rm{P}}({\rm{AP}}),V}^{{\rm{SDD}}}$ based on SDD model, which is induced by tiny charge dipoles formed in the FM_1(2)_/I interface, can be expressed by

3 $${C}_{{\rm{P}}({\rm{AP}}),V}^{{\rm{SDD}}}=eS\frac{{n}_{0,{\rm{P}}({\rm{AP}})}\xi }{\gamma V}\equiv \frac{{\alpha }_{{\rm{P}}({\rm{AP}})}}{V},$$

where *S* is a junction area, *ξ* is a characteristic screening length and ${{e}{n}}_{0,P(\text{AP})}$ is a screening charge density at the interface in P(AP) configuration. Since this screening charge acts as a serial capacitance, the total capacitance ${C}_{{\rm{P}}({\rm{AP}}),V}(f)$ at a finite bias voltage *V* in P(AP) configuration is given by

4 $${C}_{{\rm{P}}({\rm{AP}}),V}(f)={(\frac{1}{{C}_{{\rm{P}}({\rm{AP}}),V}^{{\rm{DF}}-{\rm{ZP}}}(f)}+\frac{1}{{C}_{{\rm{P}}({\rm{AP}}),V}^{{\rm{SDD}}}})}^{-1}.$$

**Figure 2:**
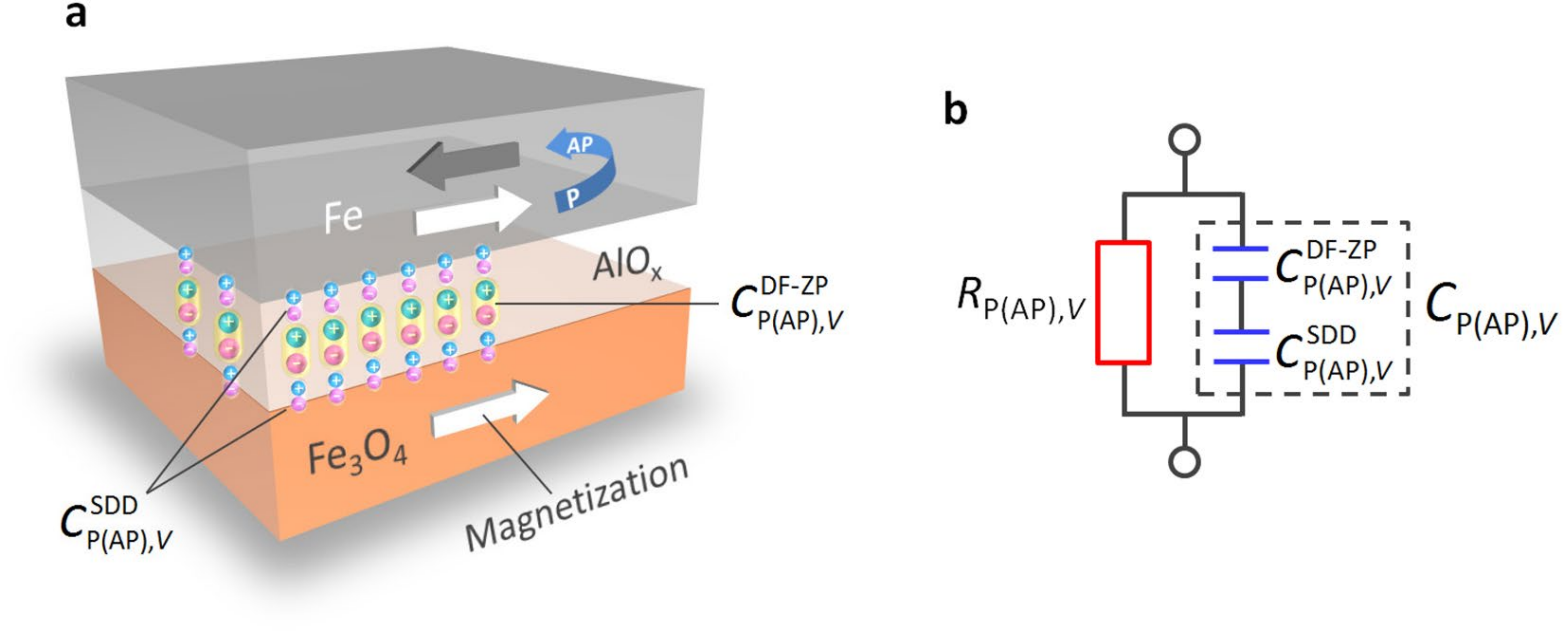
Modeling of the inverse TMC. (**a**) Schematic image of charge accumulation, contributing to ${C}_{{\rm{P}}({\rm{AP}}),V}^{\text{DF}-\text{ZP}}(f)$ and ${C}_{{\rm{P}}({\rm{AP}}),V}^{{\rm{SDD}}}$, in Fe/AlO_x_/Fe_3_O_4_. ${C}_{{\rm{P}}({\rm{AP}}),V}^{\text{DF}-\text{ZP}}(f\,)$ is described by the Debye-Fröhlich model combined with Zhang formula and parabolic barrier approximation, for the P(AP) state under the dc applied voltage *V* [equation (1)]. ${C}_{{\rm{P}}({\rm{AP}}),V}^{{\rm{SDD}}}$ is derived from the spin-dependent drift-diffusion model [equation (3)]. (**b**) Equivalent circuit of the MTJ, which is modeled by the *RC* parallel network. This model consists of the resistance ${R}_{{\rm{P}}({\rm{AP}}),V}$ and capacitance ${C}_{{\rm{P}}({\rm{AP}}),V}$. In ${C}_{{\rm{P}}({\rm{AP}}),V}$, the screening charge, contributing to ${C}_{{\rm{P}}({\rm{AP}}),V}^{{\rm{SDD}}}$, acts as a serial capacitance to ${C}_{{\rm{P}}({\rm{AP}}),V}^{\text{DF}-\text{ZP}}({\rm{f}})$.

The behavior of charge accumulation, contributing to ${C}_{{\rm{P}}({\rm{AP}}),V}^{\text{DF}-\text{ZP}}(\,f)$ and ${C}_{P(\text{AP}),V}^{{\rm{SDD}}}$, is illustrated in Fig. 2. The equivalent circuit of the MTJ is also shown. Consequently, as the inverse TMC ratio in the presence of the bias voltage is defined by

5 $${\rm{iTMC}}\,{\rm{ratio}}\,(f,V)=\frac{{C}_{{\rm{AP}},V}(f)-{C}_{{\rm{P}},V}(f)}{{C}_{{\rm{P}},V}(f)},$$

we can obtain the frequency characteristics and bias dependence of the iTMC ratio using equations (1)–(5).

### Observation of the inverse TMC

**Figure 3:**
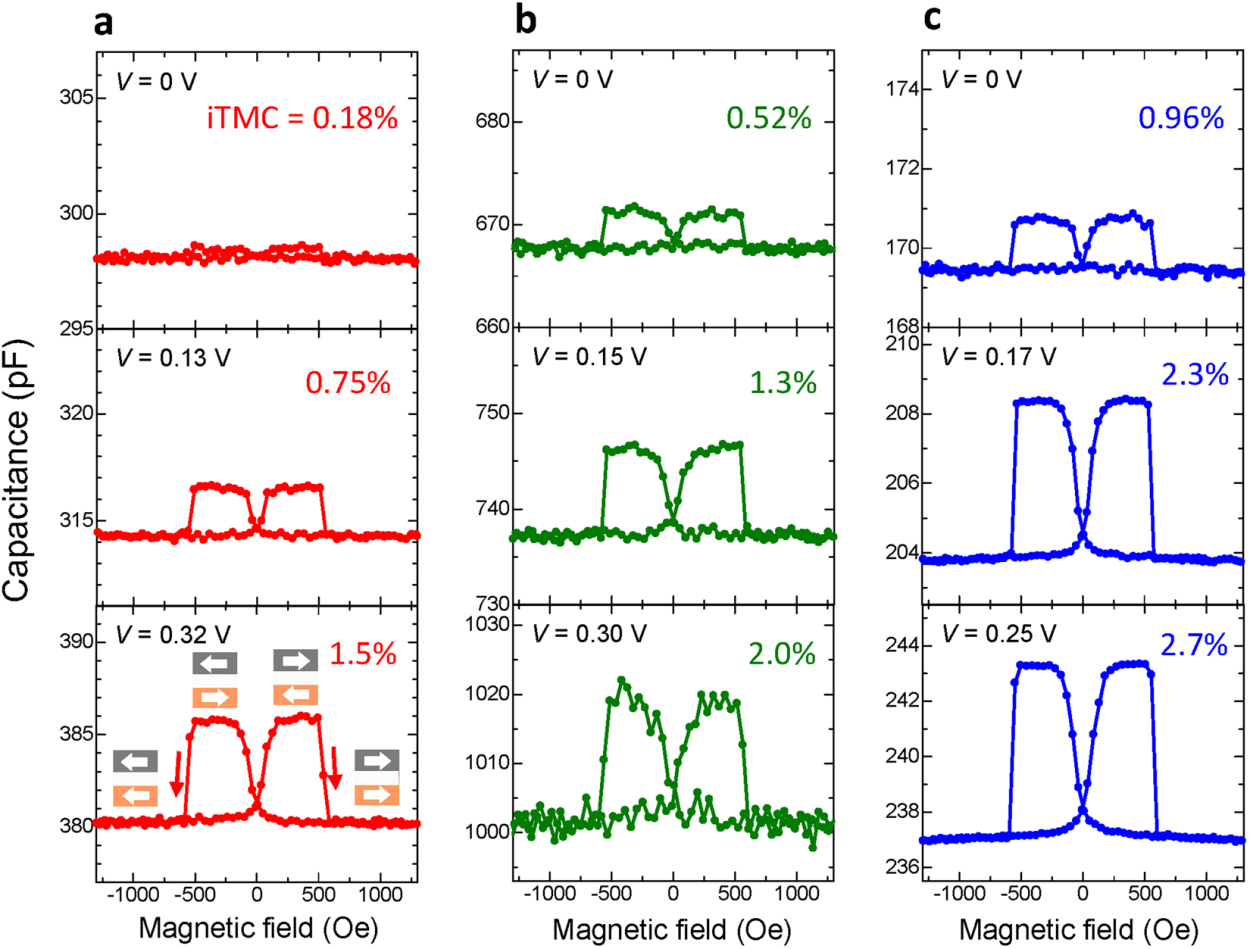
Observation of the inverse TMC. Inverse TMC effect of (**a**) sample A (*f* = 10 kHz), (**b**) sample B (*f* = 12 kHz) and (**c**) sample C (*f* = 12 kHz) at room temperature. The iTMR ratios of samples A, B and C are 1%, 7% and 15%, respectively, which are measured by a dc four probe method.

**Figure 4:**
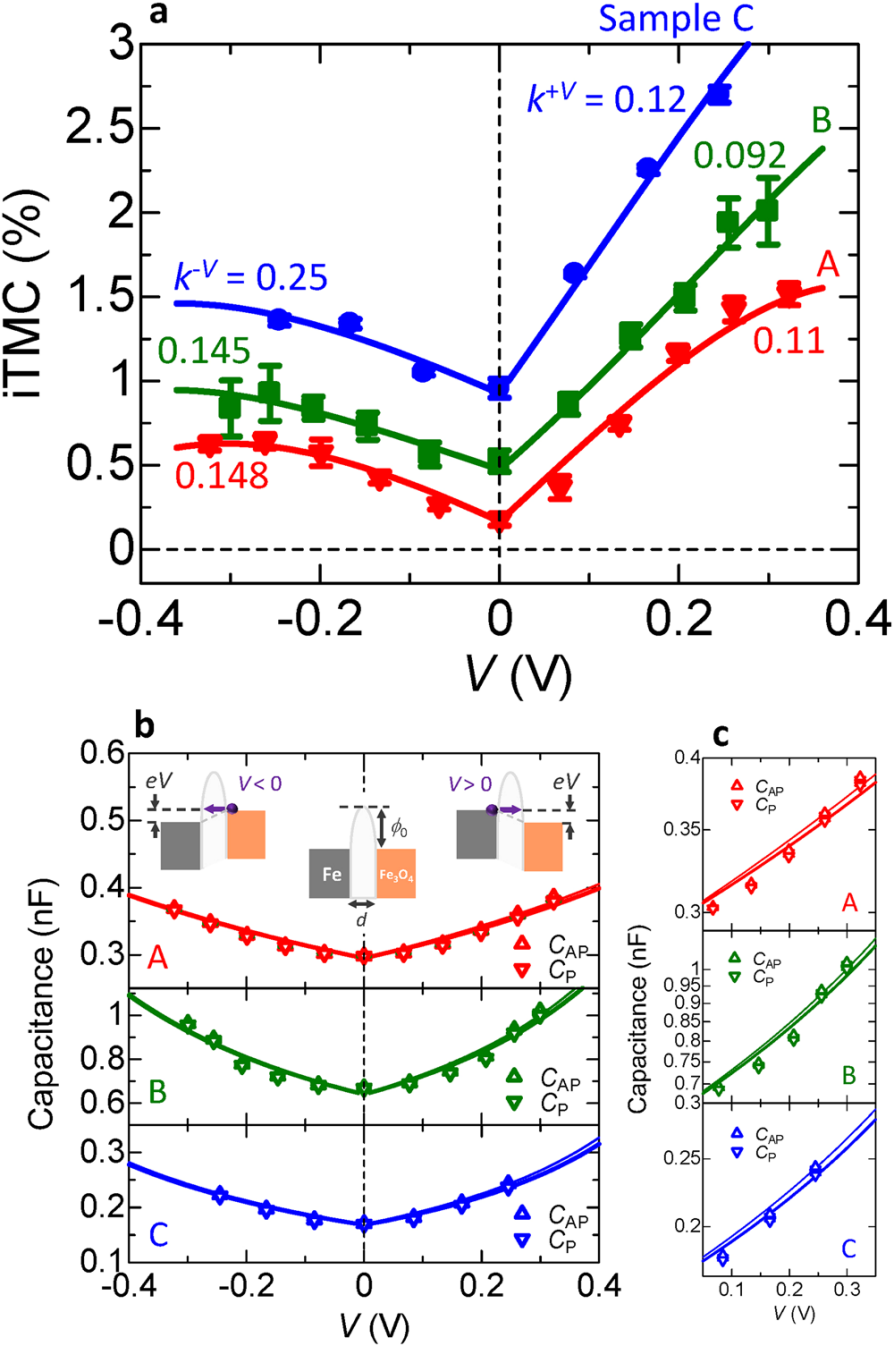
Bias dependence of the inverse TMC. Positive and negative bias dependence of (**a**) the iTMC ratio, (**b**) the capacitance *C*_P(AP)_ of the P(AP) configuration and (**c**) the log-scaled *C*_P(AP)_ in the positive bias region for samples A, B and C, respectively. *k* ^*μ*^ is the ratio ${K}_{{\rm{P}}}^{\mu }/{K}_{{\rm{AP}}}^{\mu }$, where *μ* denotes the positive or negative bias (+*V* or −*V*). The insets of the panel **b** represent the potential barriers in the absence or presence of the bias voltage *V*.

Figure 3 shows the MC response for Fe/AlO_*x*_/Fe_3_O_4_ MTJs; samples A (*f* = 10 kHz), B (*f* = 12 kHz) and C (*f* = 12 kHz), respectively. The dc bias is varied from 0 to 0.32 V. The ac voltage is set to be 35 mV_rms_. The inverse TMC effect can be clearly observed for each sample, i.e., *C*_P_ is smaller than *C*_AP_. The iTMC ratio increases from 0.18% to 1.5% with increasing the positive bias from 0 to 0.32 V for sample A, and it manifests from 1.5% to 2.7% with increasing the inverse TMR (iTMR) ratio from 1% (Sample A) to 15% (Sample C). This means that a larger iTMC ratio is observed at higher positive bias in MTJs with higher iTMR ratios. The positive and negative bias dependence of iTMC and *C*_P(AP)_ is shown in Fig. 4. The calculation of iTMC and *C*_P(AP)_ is performed using equations (1)–(5) for setting parameters shown in Supplementary Table 1. The calculation results of iTMC and *C*_P(AP)_ provide excellent fit to experimental data for each sample. The capacitance *C*_P(AP)_ increases monotonically as the bias voltage increases, for each sample. This is because the effective thickness decreases with increasing the bias voltage as described in Supplementary equation (11). Typically, the effective thickness *d*_eff_ decreases to 0.74 *d* at *V* = +0.2 V for *ϕ*_0_ = 0.17 eV and *γ* = 0.1 in sample C, where *d* is the barrier thickness in the absence of the bias voltage. The same behavior can also be found for samples A and B. This fact can be easily understood by setting *ϕ*_0_ and *γ*, which are indicated in Supplementary Table 1.

It is noted from Fig. 4a that the iTMC increases with increasing the positive and negative bias, respectively, for each sample. This can be explained by newly introduced parameters $\chi (={\tau }_{{\rm{P}},0}/{\tau }_{\text{AP},0})$ and ${k}^{\mu }(={K}_{{\rm{P}}}^{\mu }/{K}_{{\rm{AP}}}^{\mu })$, where *μ* denotes the positive or negative bias (+*V* or −*V*). The effective barrier under dc and ac electric fields can be expressed by an image-force potential^38^ and a parabolic curve, respectively. The slope of the parabolic curve is much slower than that of the image-force potential. The slower slope gives rise to a remarkable change in the relaxation time; *τ*_AP,*V*_ is more sensitive to the bias voltage than *τ*_P,*V*_ because the relaxation time in AP state is shorter than that in P state for inverse TMC. This means that *K*_AP_ is larger than *K*_P_, indicating *k* ^μ^ < 1.0. As one can see from equation (2), *τ*_AP,*V*_ becomes much shorter than *τ*_P,*V*_ at a higher *V* for both *τ*_AP,0_ < *τ*_P,0_ and *K*_AP_ > *K*_P_. This results in the enhancement of inverse TMC. These interpretations are consistent with the fitting results of *χ* = 1.01, *k* ^+*V*^ = 0.11, *k* ^−*V*^ = 0.148 for sample A, *χ* = 1.07, *k* ^+*V*^ = 0.092, *k* ^−*V*^ = 0.145 for sample B and *χ* = 1.14, *k* ^+*V*^ = 0.12, *k* ^−*V*^ = 0.25 for sample C, respectively. The enhancement of inverse TMC gives an important suggestion for practical use. As is well known, the TMR decreases with increasing the bias voltage and the improvement of *V*_half_, at which the TMR drops to half of its maximum, have become one of the key issues in the development of high-performance TMR devices^39–42^. The typical value of *V*_half_ is about 1 V in MgO-based MTJs^41^. However, this value is not sufficient for device application. Contrary to the TMR, the iTMC increases with increasing the bias voltage. This robustness in the bias voltage could be one of the advantages for future applications.

**Figure 5:**
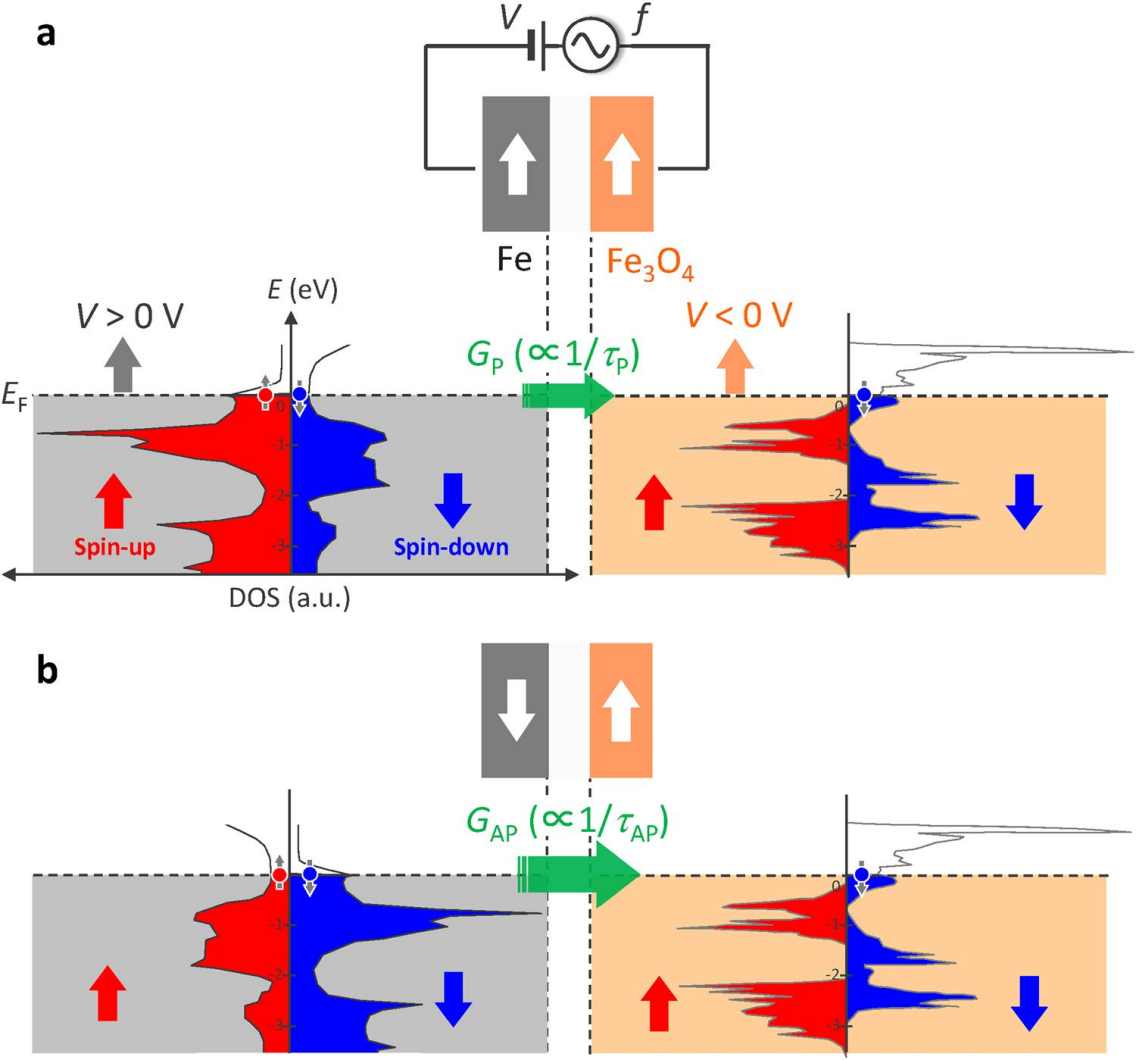
Band diagram of Fe and Fe_3_O_4_. (**a**) P and (**b**) AP configurations. The DOS of Fe is calculated by tight-binding linear muffin-tin orbital in the atomic sphere approximation (TB-LMTO-ASA) method and that of Fe_3_O_4_ is obtained from ref. 28. The Fermi level *E*_F_ is at 0 eV. The DOS in the left and right sides represents the energy band of spin-up (red) and spin-down (blue) electrons, respectively. The *E*_F_ of Fe (gray panel) goes up for *V* > 0 V and that of Fe_3_O_4_ (orange panel) goes up for *V* < 0 V, relatively to each other. The band calculation suggests that the conductance *G*_AP_ is larger than *G*_P_, in other words, the relaxation time ﻿*τ*_AP_ is shorter than *τ*_P_, indicating the appearance of inverse TMC. This inverse TMC is strongly dependent on the bias voltage *V* and its behavior is changed by the polarity of *V*. The mechanism is discussed in the main text.

From Fig. 4a, the asymmetric behavior can also be observed for the bias voltage. This is attributed to the DOS near Fermi level in Fe and Fe_3_O_4_. For the positive applied voltage, the Fermi level of Fe goes up in our device geometry. As shown in Fig. 5, there is no energy state at Fermi level for spin-up electrons of Fe_3_O_4_^28^. Therefore, spin-down electrons mainly contribute to the tunneling regime. Since the DOS of spin-down electrons for Fe is almost the same within a few 100 meV lower than *E*_F_, there is no significant change in the increasing iTMC behavior. The increase of iTMC with *V* has been explained by the above discussion based on *χ* and *k* ^*μ*^. On the other hand, for the negative bias, the Fermi level of Fe_3_O_4_ goes up relatively to that of Fe. As we focus on the P configuration [Fig. 5a], the DOS of spin-up electrons for Fe_3_O_4_ rapidly increases and that of spin-down electrons rapidly decreases within a few 100 meV lower than *E*_F_. The DOS of spin-up electrons for Fe is larger than that of spin-down electrons. These band structures bring about a remarkable increase in *G*_P_, i.e., a reduction in *τ*_P_, resulting in preventing the iTMC enhancement. Therefore, the iTMC for the positive bias is larger than that for the negative bias, which is consistent with the fitting results of *k* ^+*V*^(=0.092–0.12) < *k* ^*−V*^(=0.145–0.25), indicated in Fig. 4a.

### Large inverse TMC and its frequency response

**Figure 6:**
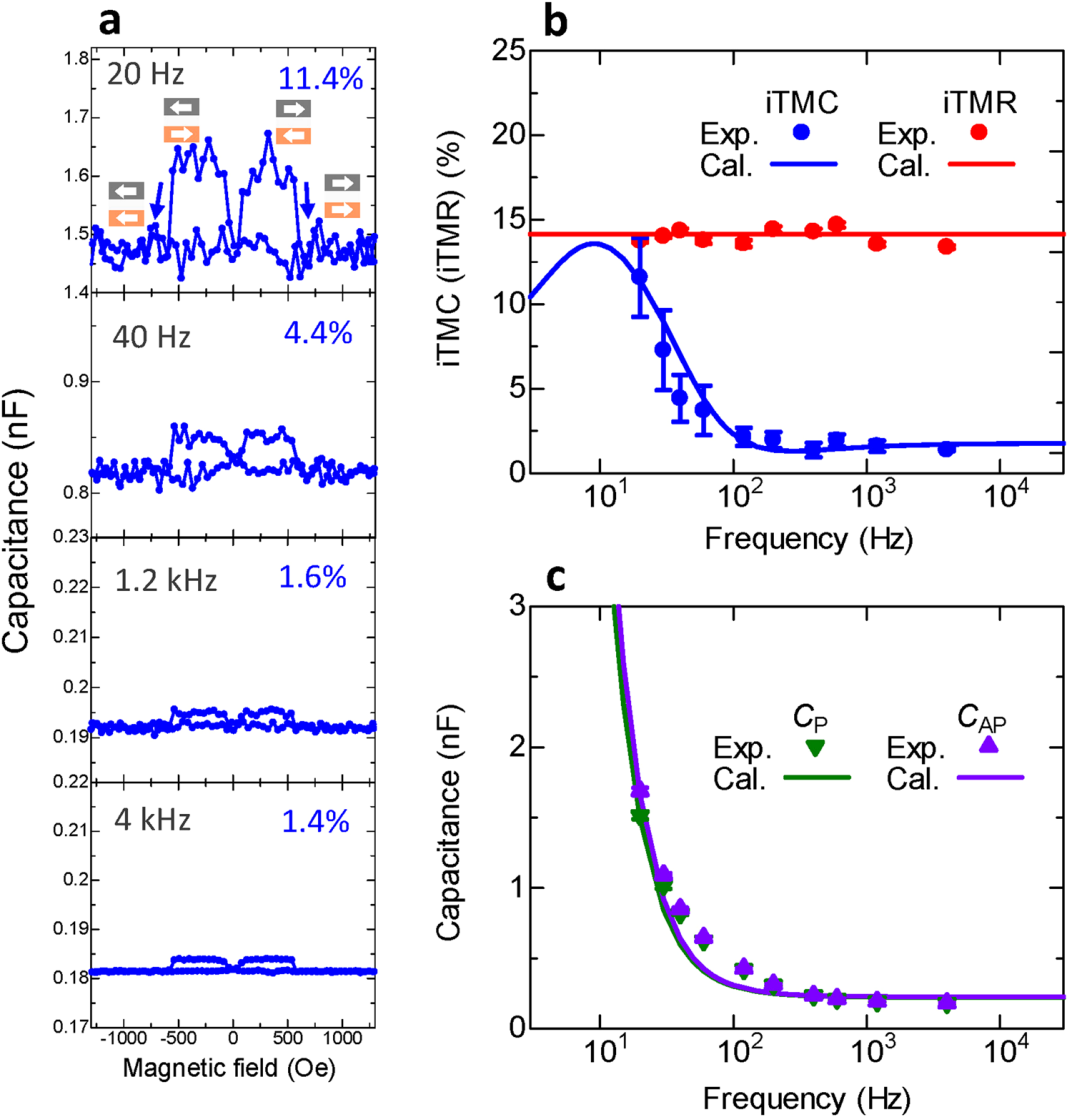
Frequency dependence of the inverse TMC. (**a**) Inverse TMC effect of Fe/AlO_*x*_/Fe_3_O_4_ MTJs with an iTMR of 15% (sample C). The frequency is 20, 40, 1.2 k and 4 kHz and the applied bias voltage is 0.09 V. The iTMC can reach 11.4 % at 20 Hz. Frequency dependence of (**b**) iTMC and iTMR and (**c**) the capacitance *C*_P(AP)_ in the P(AP) configuration. The experimental results can be well reproduced by DF model combined with Zhang formula, parabolic barrier approximation, and SDD model, described by equations (1)–(5).

We herein note that the maximum value of observed iTMC ratio is approximately 2.7% at 12 kHz for sample C, as shown in Fig. 4a. This value is smaller than expected for MTJs showing a large iTMR ratio of 15%. According to the previous results on the normal TMC, a large TMC of 155% has been observed in MTJs with a TMR of 108% at a low frequency of 200 Hz^8^. This result infers that a large iTMC could be observed in the low frequency region. Figure 6a shows the inverse TMC effect at 20, 40, 1.2 k and 4 kHz for sample C. The dc voltage is 0.09 V. A large iTMC of 11.4% is observed at a low frequency of 20 Hz and it decreases down to 1.4% with increasing the frequency. The frequency dependence of iTMC, iTMR and *C*_P(AP)_ is shown in Fig. 6b,c. The calculation of iTMC and *C*_P(AP)_ is performed by setting *C*_∞,P(AP)_ = 0.198 (0.201) nF, *C*_0,P(AP)_ = 22 (24) nF, *β*_P(AP)_ = 0.9830 (0.9872), *τ*_P,0_ = 0.06 s, *K*_P(AP)_ = 7.8 (7.2), *ϕ*_0_ = 0.18 eV, *α*_P(AP)_ = 22 (21) nC, *γ* = 0.1 and *P*_1(2)_ = 0.287 (−0.126) in equations (1)–(5). The iTMR is obtained from Julliere formula and Zhang model; the parameters are set to *K*_P(AP)_ = 7.8 (7.2) and *P*_1(2)_ = 0.428 (−0.187). The calculation results of iTMC, iTMR and *C*_P(AP)_ give a good fit to experimental data. The good fit of the frequency characteristics to our model suggests that the dynamic behavior of the dipole, formed by electrons and holes (contributing to ${C}_{{\rm{P}}({\rm{AP}}),V}^{\text{DF}-\text{ZP}}(f)$ in Fig. 2a) in the insulator, obeys the DF model; the dipole oscillates following the change of the ac electric field in the low frequency region, meanwhile it does not oscillate in the high frequency region. This means that the capacitance *C*_P(AP)_ decreases with increasing the frequency. Since the relaxation time *τ*_AP_ is shorter than *τ*_P_, the frequency characteristics of *C*_AP_ are shifted to the higher frequency region compared with those of *C*_P_. Thus, the enhancement of iTMC is observed in this shifted region, corresponding to *f* = 10–100 Hz in Fig. 6b. Here we note that *P*_1(2)_ = 0.287 (−0.126), obtained from iTMC fitting, is different from *P*_1(2)_ = 0.428 (−0.187), obtained from iTMR one. The difference of *P*_1(2)_ between iTMC and iTMR is attributed to the penetration length into the FM layers of spin-dependent carriers (electrons or holes) contributing to iTMC and iTMR. Our previous paper pointed out that the penetration length differs between normal TMC and TMR^8^. This suggests that the similar behavior can also occur in inverse TMC and TMR, i.e., the difference of the penetration length gives rise to a different *P*_1(2)_ between iTMC and iTMR.

### Bias voltage dependence of the large inverse TMC

**Figure 7:**
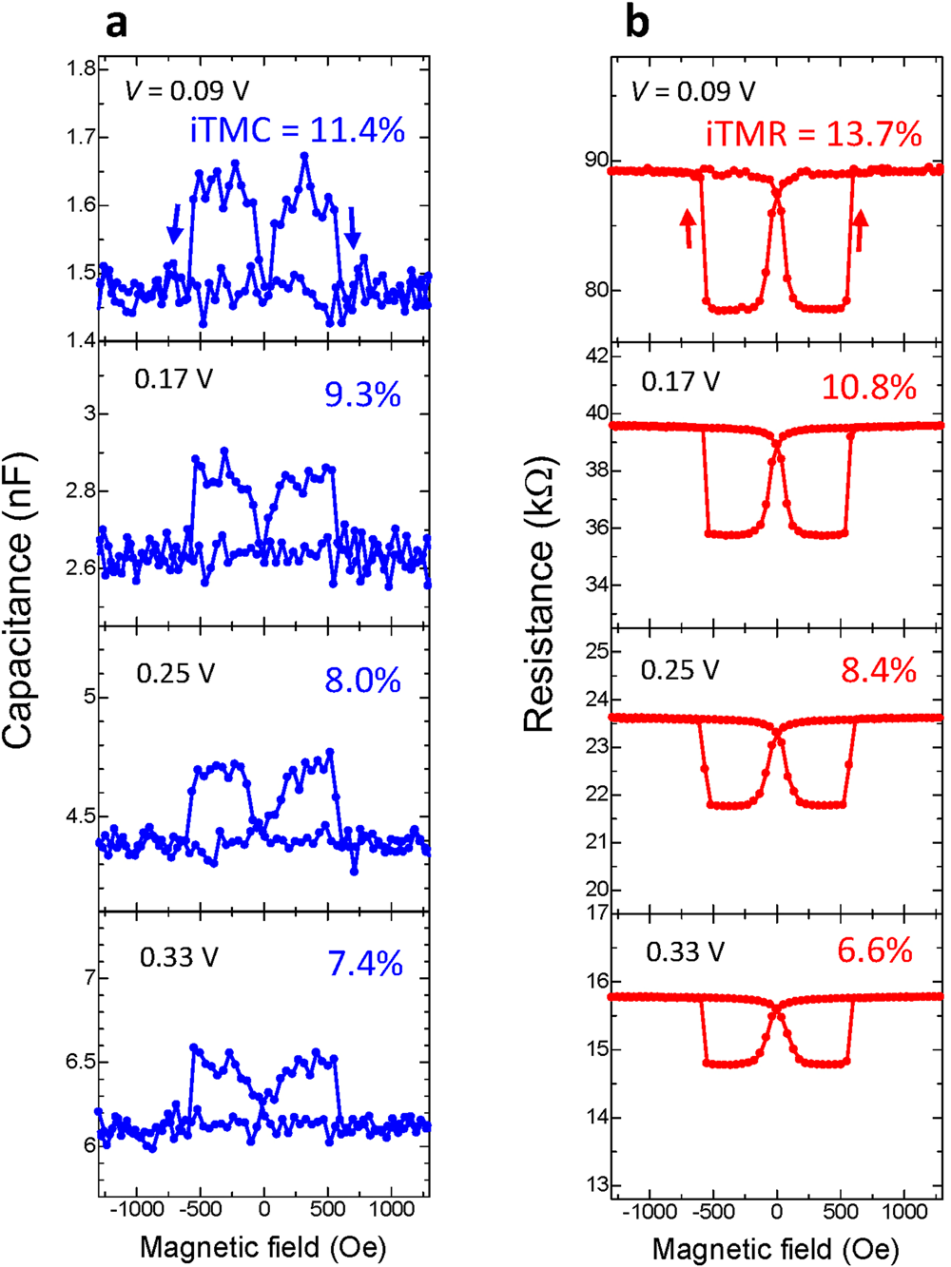
Inverse TMC and TMR. (**a**) Inverse TMC and (**b**) inverse TMR at 20 Hz in the sample C for positive bias voltages of 0.09, 0.17, 0.25 and 0.33 V.

**Figure 8:**
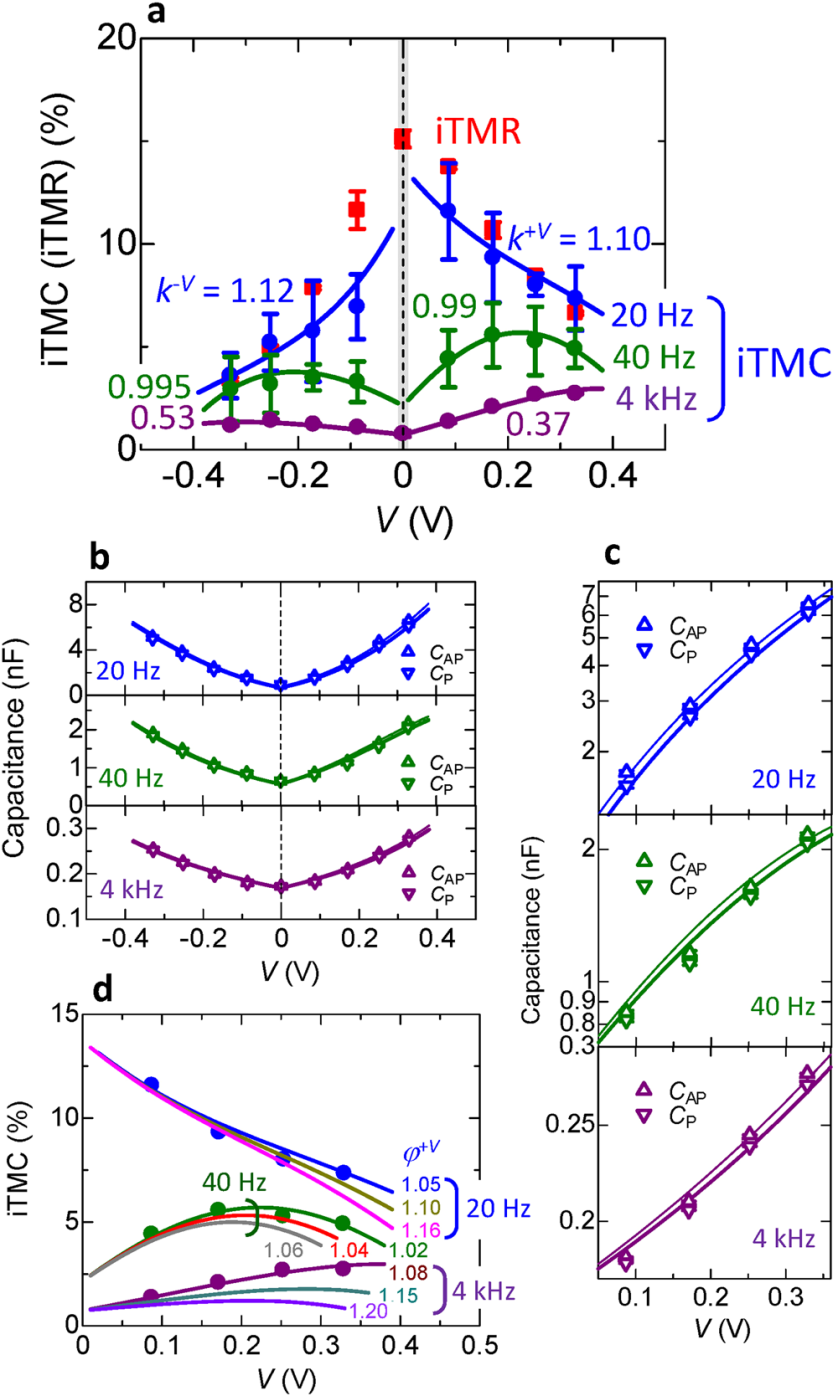
Bias dependence of large inverse TMC. Positive and negative bias dependence of (**a**) the iTMC, (**b**) the capacitance *C*_P(AP)_ of the P(AP) configuration and (**c**) the log-scaled *C*_P(AP)_ in the positive bias region at 20, 40 and 4 kHz, respectively, for sample C. The solid lines represent the fitting results calculated using the parameter, indicated in Supplementary Table 2. The iTMR at 20 Hz is also shown. (**d**) Positive bias dependence of the calculated iTMC in varying *φ* ^+V^, which is the ratio ${\alpha }_{{\rm{P}}}^{\mu }/{\alpha }_{{\rm{AP}}}^{\mu }$, described in equation (3). The best fit to experimental results is obtained by setting *φ* ^+*V*^  = 1.05, 1.02 and 1.08 at 20, 40 and 4 kHz, respectively.

Figure 7 shows the bias dependence of inverse TMC and TMR curves at 20 Hz. Contrary to the results of 12 kHz [Fig. 3c], the iTMC decreases from 11.4% to 7.4% with increasing the positive bias from 0.09 to 0.33 V. The iTMR also decreases from 13.7% to 6.6%, which exhibits the same behavior as the iTMR measured by a dc four probe method (not shown here). Although the reduction of robustness in spin polarization can be seen for both the iTMC and iTMR, the robustness of iTMC is slightly superior to that of iTMR. The positive and negative bias dependence of iTMC and *C*_P(AP)_ is plotted in Fig. 8. The frequency is 20, 40 and 4 kHz, respectively. The best fit of equations (1)–(5) is shown by the solid lines in this figure and the values of the parameters used to obtain this best fit are represented in Supplementary Table 2. The iTMR at 20 Hz is also plotted. The iTMC decreases with increasing the positive and negative bias, respectively, at 20 Hz (the data for the positive bias have been already shown in Fig. 7a). In contrast, when the frequency is 40 Hz, the iTMC increases in the low bias region and it decreases in the high bias one. At 4 kHz, the iTMC increases with increasing the bias voltage; this iTMC enhancement is also observed at 12 kHz, as shown in Fig. 4a (blue lines). These behaviors regarding the bias dependence can be characterized by the parameter *k* ^*μ*^. As discussed in Fig. 4a, the iTMC enhancement, i.e., the robustness in spin polarization, can be measured in *k* ^*μ*^ < 1.0. This means that the iTMC reduction can be seen in *k* ^*μ*^ > 1.0. The transient state is also presented at around *k* ^*μ*^~1.0. Since the fitting results reveal *k* ^+*V*^ = 1.10(>1.0) at 20 Hz, *k* ^+*V*^ = 0.99(~1.0) at 40 Hz and *k* ^+*V*^ = 0.37(<1.0) at 4 kHz, respectively, the experimental results surely support the use of the parameter k^+*V*^ to describe the robustness in spin polarization. In the case of the negative bias voltage, the similar behavior is also observed; *k* ^*−V*^ = 1.12, 0.995 and 0.53 at 20, 40 and 4 kHz, respectively.

Then, we discuss the screening charge density ${n}_{0,P(\text{AP})}^{\mu }$, contributing to spin capacitance ${C}_{{\rm{P}}({\rm{AP}}),V}^{{\rm{SDD}}}$, and the influence of ${n}_{0,{\rm{P}}(\text{AP})}^{\mu }$ on the bias dependence of iTMC. Here, *μ* denotes the positive or negative bias (+*V* or −*V*). The parameter ${\phi }^{\mu }(={\alpha }_{{\rm{P}}}^{\mu }/{\alpha }_{{\rm{AP}}}^{\mu })$ is also introduced. At 20 Hz, the excellent fit is evident using the parameters shown in Supplementary Table 2. Especially, we focus on obtaining ${\alpha }_{P(\text{AP})}^{+V}=22(21)\text{nC}$. In equation (3), the junction area is given by *S* = 10 × 10 μm^2^. The screening length *ξ* for each electrode is used as a typical value of 0.1 nm^20, 43^. This value corresponds to one-twentieth of the barrier thickness, providing a good assumption as *γ* = 0.1. Substituting these parameters in equation (3), the accumulation charge density is estimated to be ${n}_{0,P(\text{AP})}^{+V}=\,6.87(6.55)\times {10}^{23}{{\rm{cm}}}^{-3}$. This estimation is considered to be a reasonable value due to the order of Avogadro’s number *N*_A_~6.02 × 10^23^ cm^−3^. As the similar estimation is carried out using ${\alpha }_{P(\text{AP})}^{+V}=1.7(1.67)\,\text{and}\,\,0.54(0.50)\,{\rm{nC}}$ at 40 Hz and 40 kHz, the accumulation charge density can be obtained as ${n}_{0,P(\text{AP})}^{+V}=5.31(5.21)$ and 1.69(1.56) × 10^22^ cm^−3^, respectively, which are within the expected range. As for the negative bias voltage, the almost similar results are also obtained; ${n}_{0,P({\rm{AP}})}^{-V}=6.87(6.24),0.531(0.515)\,{\rm{and}}\,0.169(0.156)\times {10}^{23}\,{{\rm{cm}}}^{-3}$ at 20, 40 and 4 kHz, respectively. From these estimations, we note that ${n}_{0,{\rm{P}}}^{\mu }$ is larger than ${n}_{0,{\rm{AP}}}^{\mu }$, i.e., *φ* ^*μ*^ > 1.0 for *μ* = + *V* and *−V* at any frequency. This is attributed to the chemical potential of spin-up and spin-down electrons for the P and AP configurations in Fe/AlO_*x*_/Fe_3_O_4_. Based on the SDD model, the accumulation of spin-up electrons and the depletion of spin-down electrons remarkably take place at the interface of Fe/AlO_*x*_ and Fe_3_O_4_/AlO_*x*_ for the P configuration. This spin accumulation induces a difference in the chemical potential between spin-up and spin-down electrons. This causes a different diffusion length in each spin, giving rise to the creation of a large number of tiny charge dipoles ${n}_{0,{\rm{P}}}^{\mu }$ (contributing to ${C}_{{\rm{P}},{\rm{V}}}^{{\rm{SDD}}}$ in Fig. 2a). As a result, ${n}_{0,{\rm{P}}}^{\mu }$ is larger than ${n}_{0,{\rm{AP}}}^{\mu }$, corresponding to *φ* ^*μ*^ > 1.0. The parameter *φ* ^*μ*^ also gives a significant influence on the iTMC in a higher bias region. Figure 8d shows the bias dependence of the iTMC in varying *φ* ^+*V*^. As one can see from this figure, the remarkable enhancement in the iTMC can be measured for smaller *φ* ^+*V*^ in a higher bias region at any frequency.

### Prediction of an extremely large inverse TMC

**Figure 9:**
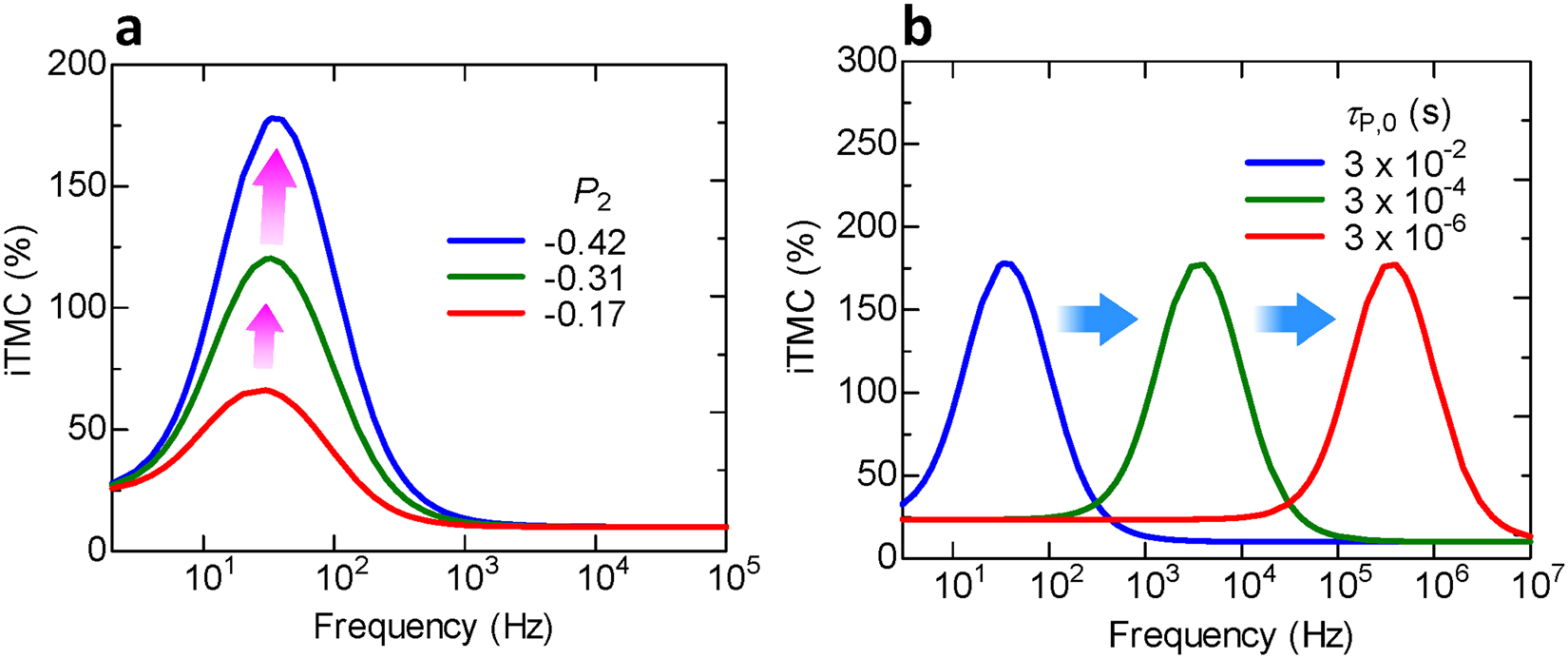
Calculated frequency dependence of extremely large inverse TMC. Frequency dependence of the iTMC in varying (**a**) *P*_2_ and (**b**) *τ*_P,0_. The calculation result predicts that the iTMC is over 150% in MTJs showing a negative TMR of −75%, which is within the realm of high-performance MTJs (﻿Se﻿﻿e ref. 45). The peak position of the iTMC can be shifted to the high frequency region in short τ_P,0_.

**Figure 10:**
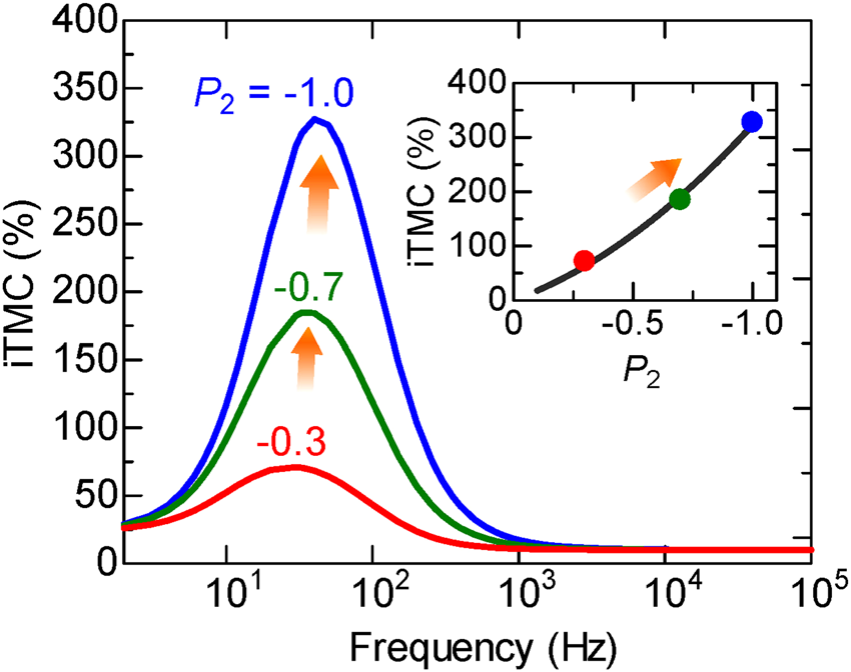
Calculated frequency dependence of iTMC in Fe_3_O_4_-based MTJs. *P*_1_ is assumed to be 0.4 for the spin polarization of Fe (See  ref. 46). The iTMC of over 300% is predicted in MTJs using Fe_3_O_4_, which could be a half metal with a *P*_2_ of −100% (blue line and plot).

Finally, we show the prediction of a large iTMC and its appearance in the high frequency region. Figure 9 shows the calculated frequency dependence of the iTMC with varying *P*_2_ and *τ*_P,0_. The parameters used in this calculation are *C*_∞,P(AP)_ = 0.20 (0.22) nF, *C*_0,P(AP)_ = 22 (28) nF, *β*_P(AP)_ = 0.9830 (0.9872), *K*_P(AP)_ = 7.8 (7.2), *ϕ*_0_ = 0.18 eV, *α*_P(AP)_ = 22 (21) nC and *γ* = 0.1. *P*_1_ is assumed to be 0.65 for the spin polarization of a commonly used CoFeB FM alloy, which is determined by point-contact Andreev reflection^44^. In Fig. 9a, *τ*_P,0_ is 0.03 s and *P*_2_ is treated as a varying parameter, with an assumed maximum value of −0.42 which is estimated experimentally for Fe_4_N at room temperature^45^. The iTMC has a maximum peak at a specific frequency. The maximum iTMC value increases from 66% to 177% when *P*_2_ increases from −0.17 to −0.42. In Fig. 9b, *τ*_P,0_ is varied, but *P*_2_ is fixed at −0.42. The maximum peak of the iTMC is shifted to a high frequency region on the order of MHz for a short *τ*_P,0_ in the μs scale. These calculations predict that a large iTMC of over 150% could be possibly observed using MTJs with a realistic *P*_1_ of 0.65 and *P*_2_ of −0.42. Furthermore, this incredibly large iTMC can be tuned from low to high frequencies by shortening *τ*_P,0_. Here we note that the Fe_3_O_4_ could be a half metal with a *P* of −100%, as described in the introduction. In this sense, it is interesting to illustrate how the peak of frequency-dependent iTMC evolves with *P*_2_. Figure 10 shows the calculated frequency dependence of iTMC with varying *P*_2_. The parameters are the same as used in Fig. 9a, except *P*_1(2)_. *P*_1_ is assumed to be 0.4 for the spin polarization of Fe^46^. The inset shows *P*_2_ dependence of the maximum iTMC. The maximum iTMC increases with increasing *P*_2_, and it reaches up to 327% for a *P*_2_ of −100%. Our observed iTMC value at −11.4% has provided us with a testing ground for our theoretical understanding. This, in turn, has led us to predict a much larger inverse TMC effect if further optimization in *P*_1_ and *P*_2_ is pursued. The effort is worthwhile as devices based on *inverse* TMC effect may find novel applications in logic circuits, energy storage devices, field-controlled oscillators and magnetic sensors.

## Methods

### Preparation of the samples

The MTJs were prepared by using an MBE system, in a chamber with a base pressure of 10^−8^ Pa, with the following layer sequence: MgO(110)/MgO(20 nm)/NiO(5 nm)/Fe_3_O_4_(60 nm)/AlO_*x*_(2–4 nm)/Fe(10 nm)/Au(30 nm). An MgO buffer layer of 20 nm was grown in a vacuum at 400 °C on an MgO(110) substrate prebaked at 800 °C. The NiO layers, which were formed by evaporation of Ni at a temperature of 300 °C in an O^*^ radical atmosphere of 4 × 10^−4^ Pa, were inserted to suppress the diffusion of Mg from the substrates. The Fe_3_O_4_ thin films were grown by reactive deposition at a temperature of 300 °C in an O_2_ atmosphere of 4 × 10^−4^ Pa. Then, the films were annealed at 600 °C for 30 min in an O_2_ atmosphere. The AlO_*x*_ insulating layers were formed by evaporation of Al_2_O_3_ at room temperature in an O_2_ atmosphere of 5 × 10^−4^ Pa. Then, the films were annealed at 150 °C in vacuum for 30 min. The Fe layers and the Au capping layers were deposited by evaporation at room temperature.

### Measurements of the inverse TMC

The frequency characteristics and the bias voltage dependence of the iTMR and iTMC for MTJs were measured by an ac four-probe method using an Agilent Technologies 4284A LCR meter at room temperature. The schematic of the measurement setup is shown in Fig. 1a. The frequency was ranged from 20 Hz to 1 MHz and the magnetic field is applied along the [−110] direction up to 1.4 kOe.

## Electronic supplementary material

Supplementary Information
