# Supplementary material for: Inverse Tunnel Magnetocapacitance in Fe/Al-oxide/Fe3O4
Source: Sci Rep. 2017 Jun 1;7:2682. doi: 10.1038/s41598-017-02361-4 (PMC5454010; doi:10.1038/s41598-017-02361-4)
Supplement: Supplementary file 1 — Supplementary Information [file 41598_2017_2361_MOESM1_ESM.pdf]

Inverse Tunnel Magnetocapacitance in Fe/Al-oxide/Fe_3O_4

Supplementary Information

Hideo Kaiju[1], Taro Nagahama[2], Shun Sasaki[2], Toshihiro Shimada[2], Osamu Kitakami[3],

Takahiro Misawa[1], Masaya Fujioka[1], Junji Nishii[1] and Gang Xiao[4]

[1]Research Institute for Electronic Science, Hokkaido University, Sapporo, Hokkaido 001-0020, Japan

[2]Graduate School of Engineering, Hokkaido University, Sapporo, Hokkaido 060-8628, Japan

[3]Institute of Multidisciplinary Research for Advanced Materials, Tohoku University, Sendai, Miyagi 980-8577, Japan

[4]Department of Physics, Brown University, Providence, RI 02912, USA

Correspondence and requests for materials should be addressed to H. K. (email: kaiju@es.hokudai.ac.jp).

In this Supplementary Information section, we present a detailed derivation of the inverse TMC and provide the parameters used in the calculation of iTMC-*V* curves shown in the main text.

Derivation of the inverse TMC. The calculation of the inverse TMC is performed using DF model (combined with Zhang formula and parabolic barrier approximation) and SDD model. The DF model is a useful tool for the calculation of dynamic dielectric constant in a variety of insulating solid and liquid systems[1–4]. This model can also be applied to the inverse TMC in MTJs, because it has been successful in accounting for the normal TMC in dielectric-based spintronic devices, such as MgO-based MTJs and FeCo-MgF nanogranular films[5,6]. Based on the model[7], the complex dielectric constant ε^* can be generally represented by

$$\varepsilon^* = \varepsilon_\infty + \frac{\varepsilon_0 - \varepsilon_\infty}{1+(i\omega\tau)^\beta}\ , \tag{1}$$

where ε_∞ and ε_0 are the high-frequency and static dielectric constants, ω is the angular frequency and τ is the relaxation time. The exponent β, indicating the distribution of relaxation time, is between 0 and 1. Since the real part of ε^* is proportional to the capacitance, the capacitance $C^{\mathrm{DF}}_{\mathrm{P(AP)}}(f)$ as a function of frequency f for the P(AP) configuration in MTJs can be expressed by

$$C^{\mathrm{DF}}_{\mathrm{P(AP)}}(f) = \mathrm{Re}\left[C_{\infty,\,\mathrm{P(AP)}} + \frac{C_{0,\,\mathrm{P(AP)}} - C_{\infty,\,\mathrm{P(AP)}}}{1+(i2\pi f\tau_{\mathrm{P(AP)}})^{\beta_{\mathrm{P(AP)}}}}\right], \tag{2}$$

where $C_{\infty,\,\mathrm{P(AP)}}$ and $C_{0,\,\mathrm{P(AP)}}$ are the high-frequency and static capacitances, $\tau_{\mathrm{P(AP)}}$ is the relaxation time and $\beta_{\mathrm{P(AP)}}$ is the exponent showing the distribution of relaxation time, respectively, for the P(AP) configuration. After a straightforward calculation of equation (2), we can obtain

$$C^{\mathrm{DF}}_{\mathrm{P(AP)}}(f) = C_{\infty,\,\mathrm{P(AP)}} + \frac{C_{0,\,\mathrm{P(AP)}} - C_{\infty,\,\mathrm{P(AP)}}}{2}\left[1 - \frac{\sinh[\beta_{\mathrm{P(AP)}}\ln(2\pi f\tau_{\mathrm{P(AP)}})]}{\cosh[\beta_{\mathrm{P(AP)}}\ln(2\pi f\tau_{\mathrm{P(AP)}})] + \cos(\beta_{\mathrm{P(AP)}}\pi/2)}\right]. \tag{3}$$

According to Julliere formula[8], the relation between τ_{P} and τ_{AP} in $\mathrm{FM_1}$/insulator/$\mathrm{FM_2}$ ($P_1 > 0$ and $P_2 < 0$) is given by

$$\tau_{\mathrm{AP}} = \frac{1-|P_1||P_2|}{1+|P_1||P_2|}\tau_{\mathrm{P}}\ . \tag{4}$$

Therefore, under no bias voltage, as the inverse TMC ratio is defined by

$$\text{iTMC ratio}(f) = \frac{C_{\text{AP}}^{\text{DF}}(f) - C_{\text{P}}^{\text{DF}}(f)}{C_{\text{P}}^{\text{DF}}(f)}, \tag{5}$$

we can find the frequency characteristics of the iTMC ratio by substituting $C_{\infty,\,\text{P(AP)}}$, $C_{0,\,\text{P(AP)}}$, $\beta_{\text{P(AP)}}$, τ_{P} and $P_{1(2)}$ in equations (3)−(5).

In addition to this procedure, the following three models are taken into account to describe the inverse TMC at a finite bias voltage; i) Zhang formula, ii) parabolic barrier approximation and iii) SDD model. According to Zhang's theory[9], the tunnel conductance in FM_1/insulator/FM_2 strongly depends on the bias voltage, especially within the order of a few hundred millivolts, due to hot electrons producing spin excitations. The conductance $G_{\text{P(AP)},V}$ at a finite bias voltage V in the P(AP) configuration can be expressed by $G_{\text{P(AP)},V} = G_{\text{P(AP)},0}\left(1+K_{\text{P(AP)}}V\right)$, where $G_{\text{P(AP)},0}$ is a conductance at zero bias in the P(AP) configuration and $K_{\text{P(AP)}}$ is a parameter determined by Curie temperatures of FM_1 and FM_2, the DOS of itinerant electrons in FM_1 and FM_2, and direct and spin-dependent transfers and spin quantum number within the framework of the transfer Hamiltonian in the system of FM_1/insulator/FM_2. The $K_{\text{P(AP)}}$ is assumed to be an adjustable parameter in our calculation. Since the assumption that the relaxation time is inversely proportional to the conductance can give a good agreement with experimental data[5,6], the relaxation time $\tau_{\text{P(AP)},V}$ with applied voltage can be written by

$$\tau_{\text{P(AP)},V} = \frac{1}{1+K_{\text{P(AP)}}V}\tau_{\text{P(AP)},0}, \tag{6}$$

where $\tau_{\text{P(AP)},0}$ is the relaxation time at zero bias voltage in the P(AP) configuration; equation (4) is modified to $\tau_{\text{AP},0} = \tau_{\text{P},0}(1-|P_1||P_2|)/(1+|P_1||P_2|)$. Therefore, $\tau_{\text{P(AP)}}$ in equaiton (3) should be replaced to $\tau_{\text{P(AP)},V}$, described by equation (6), under the application of the bias voltage.

Next, we consider the bias voltage dependence of the effective barrier thickness, which contributes to the measured capacitance. The potential profile in the barrier is assumed to be a parabolic function, which is known as a good approximation to describe tunneling process, such as ac tunneling transport explained by DF model[5,6] and fluctuation-induced tunneling (FIT) proposed by Sheng[10]. In this parabolic barrier approximation, the potential function $\phi(u)$ under the bias voltage V can be expressed by $\phi(u) = 4\phi_0(1-u)u + eVu$, where $u = x/d$ is the reduced spatial variable, x is the distance from the surface of the one side electrode, d is the barrier thickness, ϕ_0 is the barrier height in the absence of the bias voltage and e is the electron charge.

Since the solution of $\phi(u)=eV$ is $u_1 = eV/4\phi_0$ and $u_2 = 1$ (for $u_1 < u_2$), the effective barrier thickness $d_{\rm eff}$ can be represented by

$$d_{eff} = (1 - \frac{eV}{4\phi_0})d\,. \tag{7}$$

Therefore, from equations (3), (6) and (7), the capacitance $C^{\rm DF\text{-}ZP}_{\rm P(AP),\mathit{V}}(f)$ at a finite applied voltage in the P(AP) configuration, based on Zhang model and parabolic barrier approximation, can be written by

$$C^{\rm DF-ZP}_{\rm P(AP),\mathit{V}}(f) = \frac{1}{1 - eV/4\phi_0}\left[C_{\infty,\rm\, P(AP)} + \frac{C_{0,\rm\, P(AP)} - C_{\infty,\rm\, P(AP)}}{2}\left(1 - \frac{\sinh[\beta_{\rm P(AP)}\ln(2\pi f\tau_{\rm P(AP),\mathit{V}})]}{\cosh[\beta_{\rm P(AP)}\ln(2\pi f\tau_{\rm P(AP),\mathit{V}})] + \cos(\beta_{\rm P(AP)}\pi/2)}\right)\right]. \tag{8}$$

Finally, we incorporate spin capacitance caused by the difference between spin-up and spin-down diffusion length, described by SDD model[11]. According to this model, accumulation of minority spins and depletion of majority spins, taking place at the interface between the ferromagnetic layer and insulator, form a tiny screening charge dipole. This dipole gives rise to an addition serial capacitance, which corresponds to spin capacitance. The screening charge density in P(AP) configuration is given by $en_{\rm P(AP)}(x) = en_{0,\rm P(AP)}\exp(-x/\xi)$, where x is the distance from the interface between one-side electrode and insulator, ξ is a characteristic screening length and $en_{0,\rm P(AP)}$ is a screening charge density at the interface in P(AP) configuration. Since the spin capacitance in P(AP) configuration can be expressed by $C^{\rm SDD}_{\rm P(AP),\mathit{V}} = \Delta Q_{\rm P(AP)}/\Delta V$, where $\Delta Q_{\rm P(AP)}$ is the screening charge for P(AP) configuration and ΔV is the electrical potential difference applied in the charging space, it is given by $C^{\rm SDD}_{\rm P(AP),\mathit{V}} = eSn_{\rm P(AP)}(x)dx/dV(x)$, where S is a junction area and $V(x)$ is an electrical potential as a function of x in the charging space, i.e., $V(x) = V_{\rm eff}\exp(-x/\xi)$, where $V_{\rm eff}$ is an effective applied voltage. $V_{\rm eff}$ is assumed to be γV, where γ is an adjustable positive parameter of much smaller than 1.0. From these equations, we can find a simple formula:

$$C^{\rm SDD}_{\rm P(AP),\mathit{V}} = eS\frac{n_{0,\rm\, P(AP)}\xi}{\gamma V} \equiv \frac{\alpha_{\rm P(AP)}}{V}\,. \tag{9}$$

Since this screening charge acts as a serial capacitance, V in equations (6)–(8) should be modified into $(1-\gamma)V$. Therefore, these equations are replaced to

$$\tau_{\mathrm{P(AP)},V} = \frac{1}{1+K_{\mathrm{P(AP)}}(1-\gamma)V}\tau_{\mathrm{P(AP)},0}, \tag{10}$$

$$d_{eff} = \left(1-\frac{e(1-\gamma)V}{4\phi_0}\right)d, \tag{11}$$

$$C_{\mathrm{P(AP)},V}^{\mathrm{DF-ZP}}(f) = \frac{1}{1-e(1-\gamma)V/4\phi_0}\left[C_{\infty,\,\mathrm{P(AP)}} + \frac{C_{0,\,\mathrm{P(AP)}}-C_{\infty,\,\mathrm{P(AP)}}}{2}\left(1-\frac{\sinh[\beta_{\mathrm{P(AP)}}\ln(2\pi f\tau_{\mathrm{P(AP)},V})]}{\cosh[\beta_{\mathrm{P(AP)}}\ln(2\pi f\tau_{\mathrm{P(AP)},V})]+\cos(\beta_{\mathrm{P(AP)}}\pi/2)}\right)\right]. \tag{12}$$

From these procedures, the capacitance $C_{\mathrm{P(AP)},V}(f)$ at a finite bias voltage V in P(AP) configuration is given by

$$C_{\mathrm{P(AP)},V}(f) = \left(\frac{1}{C_{\mathrm{P(AP)},V}^{\mathrm{DF-ZP}}(f)}+\frac{1}{C_{\mathrm{P(AP)},V}^{\mathrm{SDD}}}\right)^{-1}. \tag{13}$$

The behavior of charge accumulation, contributing to $C_{\mathrm{P(AP)},V}^{\mathrm{DF\text{-}ZP}}(f)$ and $C_{\mathrm{P(AP)},V}^{\mathrm{SDD}}$, is illustrated in Fig. 2 in the main text. The equivalent circuit of the MTJ is also shown. Consequently, as the iTMC ratio in the presence of the applied voltage is defined by

$$\text{iTMC ratio}(f,V) = \frac{C_{\mathrm{AP},V}(f)-C_{\mathrm{P},V}(f)}{C_{\mathrm{P},V}(f)}, \tag{14}$$

we can obtain the frequency characteristics and bias dependence of the iTMC ratio using equations (9)−(14).

Parameters used in the calculation. The parameters used in the calculation of iTMC-V curves shown in Figs. 4 and 8 in the main text are listed in Tables 1 and 2, respectively.

Table 1 Parameters used in the calculation of iTMC-V curves shown in Fig. 4 in the main text.

Positive bias

Sample	$C_{0,P(AP)}$ (nF)	$C_{\infty,P(AP)}$ (nF)	$\tau_{P,0}$ (s)	$\beta_{P(AP)}$	$K_{P(AP)}$ (V^{-1})	ϕ_0 (eV)	$\alpha_{P(AP)}$ (nC)	γ	$P_{1(2)}$
**A**	78(79)	0.295(0.296)	0.075	0.9875(0.9880)	8.9(83)	0.20	0.60(0.57)	0.1	0.40(−0.013)
**B**	89(96)	0.640(0.643)	0.075	0.9875(0.9880)	10(109)	0.17	4.0(3.9)	0.1	0.40(−0.085)
**C**	60(69)	0.168(0.170)	0.075	0.9875(0.9880)	7.8(64)	0.17	1.1(1.0)	0.1	0.39(−0.17)

Negative bias

Sample	$C_{0,P(AP)}$ (nF)	$C_{\infty,P(AP)}$ (nF)	$\tau_{P,0}$ (s)	$\beta_{P(AP)}$	$K_{P(AP)}$ (V^{-1})	ϕ_0 (eV)	$\alpha_{P(AP)}$ (nC)	γ	$P_{1(2)}$
**A**	78(79)	0.295(0.296)	0.075	0.9875(0.9880)	8.9(60)	0.21	0.60(0.57)	0.1	0.40(−0.013)
**B**	89(96)	0.640(0.643)	0.075	0.9875(0.9880)	10(69)	0.19	4.0(3.7)	0.1	0.40(−0.085)
**C**	60(69)	0.168(0.170)	0.075	0.9875(0.9880)	7.8(31)	0.20	1.1(1.0)	0.1	0.39(−0.17)

Table 2 Parameters used in the calculation of iTMC-*V* curves shown in Fig. 8 in the main text.

Positive bias

f (Hz)	$C_{0,P(AP)}$ (nF)	$C_{\infty,P(AP)}$ (nF)	$\tau_{P,0}$ (s)	$\beta_{P(AP)}$	$K_{P(AP)}$ (V^{-1})	ϕ_0 (eV)	$\alpha_{P(AP)}$ (nC)	γ	$P_{1(2)}$
**20**	22(24)	0.1975(0.2010)	0.06	0.9830(0.9880)	7.8(7.1)	0.18	22.0(21.0)	0.1	0.30(−0.13)
**40**	35(37)	0.1680(0.1691)	0.06	0.9500(0.9600)	4.2(4.3)	0.14	1.70(1.67)	0.1	0.30(−0.13)
**4k**	50(57)	0.1680(0.1691)	0.06	0.9875(0.9880)	11(30)	0.16	0.54(0.50)	0.1	0.30(−0.13)

Negative bias

f (Hz)	$C_{0,P(AP)}$ (nF)	$C_{\infty,P(AP)}$ (nF)	$\tau_{P,0}$ (s)	$\beta_{P(AP)}$	$K_{P(AP)}$ (V^{-1})	ϕ_0 (eV)	$\alpha_{P(AP)}$ (nC)	γ	$P_{1(2)}$
**20**	22(24)	0.1975(0.2010)	0.06	0.9830(0.9872)	7.8(6.9)	0.24	22.0(20.0)	0.1	0.28(−0.12)
**40**	35(37)	0.1680(0.1691)	0.06	0.9500(0.9600)	2.4(2.5)	0.12	1.70(1.65)	0.1	0.30(−0.13)
**4k**	50(57)	0.1680(0.1691)	0.06	0.9875(0.9880)	11(21)	0.18	0.54(0.50)	0.1	0.30(−0.13)

References

1. Gorter, C. J. & Kronig, R. de L. On the theory of absorption and dispersion in paramagnetic and dielectric media. *Physica* **3**, 1009−1020 (1936).
2. Casimir, H. B. G. & du Pre, F. K. Note on the thermodynamic interpretation of paramagnetic relaxation phenomena. *Physica* **5**, 507−511 (1938).
3. Zener, C. Internal friction in solids. *Phys. Rev.* **53**, 90−99 (1938).
4. Cole, K. S. & Cole, R. H. Dispersion and absorption in dielectrics. *J. Chem. Phys.* **9**, 341−351 (1941).
5. Kaiju, H., Takei, M., Misawa, T., Nagahama, T., Nishii, J. & Xiao, G. Large magnetocapacitance effect in magnetic tunnel junctions based on Debye-Fröhlich model. *Appl. Phys. Lett.* **107**, 132405 (2015).
6. Kobayashi, N., Masumoto, H., Takahashi, S. & Maekawa, S. Giant dielectric and magnetoelectric responses in insulating nanogranular films at room temperature. *Nat. Commun.* **5**, 4417 (2014).
7. Fröhlich, H. *Theory of dielectrics : dielectric constant and dielectric loss* (Clarendon, Oxford, 1958).
8. Julliere, M. Tunneling between ferromagnetic films. *Phys. Lett.* **54A**, 225−226 (1975).
9. Zhang, S., Levy, P. M., Marley, A. C. & Parkin, S. S. P. Quenching of magnetoresistance by hot electrons in magnetic tunnel junctions. *Phys. Rev. Lett.* **79**, 3744−3747 (1997).
10. Sheng, P. Fluctuation-induced tunneling conduction in disordered materials. *Phys. Rev.* B **21**, 2180−2195 (1980).
11. Lee, T.-H. & Chen, C.-D. Probing spin accumulation induced magnetocapacitance in a single electron transistor. *Sci. Rep.* **5**, 13704 (2015).
